# Fold change based approach for identification of significant network markers in breast, lung and prostate cancer

**DOI:** 10.1049/iet-syb.2018.0012

**Published:** 2018-10-01

**Authors:** Richa K. Makhijani, Shital A. Raut, Hemant J. Purohit

**Affiliations:** ^1^ Department of Computer Science and Engineering Visvesvaraya National Institute of Technology Nagpur (MS) 440010 India; ^2^ Environmental Genomics Division National Environmental Engineering Research Institute Nagpur (MS) 440020 India

**Keywords:** cancer, proteins, RNA, bioinformatics, statistical analysis, genetics, molecular biophysics, ontologies (artificial intelligence), lung, cancer network, PPI data, gene sets, multiple cancers, Gene Ontology functional enrichment, prostate cancer, gene expression data, RNA‐seq platforms, protein–protein interaction network, DEG, microarray data, RNA‐seq data, cancer types, lung cancer, diseases, breast cancer, network markers, differentially expressed genes, fold change based approach, CFinder, statistical methods

## Abstract

Cancer belongs to a class of highly aggressive diseases and a leading cause of death in the world. With more than 100 types of cancers, breast, lung and prostate cancer remain to be the most common types. To identify essential network markers (NMs) and therapeutic targets in these cancers, the authors present a novel approach which uses gene expression data from microarray and RNA‐seq platforms and utilises the results from this data to evaluate protein–protein interaction (PPI) network. Differentially expressed genes (DEGs) are extracted from microarray data using three different statistical methods in R, to produce a consistent set of genes. Also, DEGs are extracted from RNA‐seq data for the same three cancer types. DEG sets found to be common in both platforms are obtained at three fold change (FC) cut‐off levels to accurately identify the level of change in expression of these genes in all three cancers. A cancer network is built using PPI data characterising gene sets at log‐FC (LFC)>1, LFC>1.5 and LFC>2, and interconnection between principal hub nodes of these networks is observed. Resulting network of hubs at three FC levels highlights prime NMs with high confidence in multiple cancers as validated by Gene Ontology functional enrichment and maximal complete subgraphs from CFinder.

## 1 Introduction

Cancer gene discovery is an important challenge clinically and computationally in a comprehensive genetic context, where a wide variety of omics data are available. Over the last decade, an extensive research is headed towards close‐fitting the divergence of molecular and cellular processes in case of human cancer. Different cancer types have been investigated in studies using different clinical or in‐silico data types such as gene expression using DNA microarray and RNA‐seq technology, pathway and regulatory data, protein–protein interaction (PPI) data and their meta‐analysis [1–5]. Numerous researchers have published interesting results in the identification of biomarkers as a result of bioinformatics analysis, which can yield deep insight in understanding the biological processes of cancer oncology [6, 7]. A high number of deaths attributable to breast cancer in women, prostate cancer in men, and lung cancer across both men and women, are observed worldwide (Cancer facts and figures 2017 and WHO cancer country profiles). Hence, detection of differentially expressed genes (DEGs) is essential to understand the complex functional changes that occur in the disease. Enormous cancer data from microarray and RNA‐seq technology is available in public on Gene Expression Omnibus (GEO) [8] and The Cancer Genome Atlas (TCGA) [9]. Recent experimentation involves analysis of cancer data from both these platforms to compare or combine their results for gene identification. Such cross‐platform comparison produces high reproducibility among biological replicates [10]. To focus on the difference, rather than similarity, between RNA‐seq and microarray technologies, a comparison was presented using RNA samples from a human T‐cell activation experiment [11]. Benefits of RNA‐seq were identified which are, a broader dynamic range for detection of more number of DEGs with higher fold change (FC), a high correlation between gene expression profiles and detection of low abundance transcripts. Also, RNA‐seq was found to be more accurate in identifying DEGs relative to microarray analysis [12]. Still, microarrays continue to be a more common choice of researchers in gene profiling experiments due to RNA‐seq being new, expensive and complex in data storage and analysis. A similar analysis on both platforms produced strongly concordant and highly correlated results [13]. A common set of DEGs evident in three cancers was derived using such cross‐platform analysis and heterogeneity across the cancers was identified [14]. Recently, network‐based studies are performed on complex diseases including cancer, to unravel the dysregulation of pathways and processes involved [15]. In addition to such cross‐platform identification of genes, there is a need to identify the implications of the level of FC on the disease network, at the interaction level. Capturing statistical variability at the genetic level along with biological variability in terms of FC helps in identifying the relationship between hub nodes of PPI network at the protein level.

Hence, we present a FC‐based approach to extract DEGs from microarray and RNA‐seq datasets of said three cancer types at three different FC levels. We then evaluate the effects of FC at the interaction level with a prime objective to discover a FC‐based signature of common network markers (NMs) in multiple cancer types (breast, lung and prostate). A network shared by the three cancers is constructed with the view of FC‐based regulation and relation of genes. The findings demonstrate the usefulness of our approach in understanding the biological implications of FC in the pathways of three cancers.

## 2 Materials and methods

### 2.1 Datasets

We used three cancer datasets: breast, lung and prostate, for both microarray and RNA‐seq platforms from GEO. Accession numbers (number of tumour samples/number of normal samples) are GSE45827 (149/11) [16], GSE48984 (3/9) [17], GSE26910 (6/6) [18], GSE19804 (60/60) [19], GSE10072 (57/50) [20], GSE55945 (12/7) [21], GSE26910 (6/6) for microarray data and GSE62944 (2120/213) [22] for RNA‐seq data. Total samples analysed are 442 (293 tumour/149 normal) for microarray and 2333 (2120 tumour /213 normal) for RNA‐seq. Robust multichip average was used for expression normalisation of microarray data as it showed good differential change detection, stable variance and less number of false positives [23].

### 2.2 Identification of DEG sets

#### 2.2.1 Microarray data

Selection of an appropriate method for extracting best results from microarray analysis is challenging due to the arguments on their inconsistency. It is thus recommended to acknowledge DEGs that lie within an intersection of DEG sets obtained by different methods, preferably, linear modelling methods for microarray analysis (LIMMA), significance analysis of microarray (SAM) and T‐test [24]. Thus, to address the issue of result inconsistency in extraction of DEGs, three different methods were used. These are LIMMA [25], SAM [26] and FC rank ordering statistic (FCROS) [27]. LIMMA which uses emperical Bayes statistic shows advantages in terms of statistical power, false‐positive rate, execution time and ease of use [28, 29]. SAM is a repeated permutation‐based method which assigns a score to each gene on the basis of change in expression relative to the standard deviation. As our objective was to emphasise FC for identification of DEGs, we used a rank‐based approach, FCROS, that associates a statistic with the ranks of the FC values for each gene, and then uses the resulting probability to identify the DEGs within an error level. It shows advantages of being deterministic, fast and it overcomes multiple testing problems associated with microarray datasets. It is known that compared to methods using mere statistical parameters, results obtained from FC‐based methods are more reproducible and biologically relevant, irrespective of the technology used [27]. Therefore, FC was chosen as a crucial condition for gene selection.

**Fig. 1:**
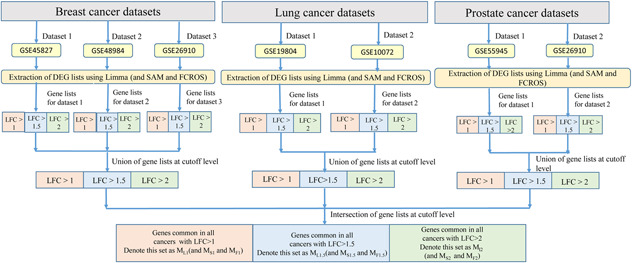
Pipeline for identification of DEGs in three cancer types from microarray data using LIMMA (and SAM and FCROS)

Fig. 1 shows complete pipeline for DEG extraction from microarray data. The parameters used for the three methods are listed as, LIMMA: *P*‐value<0.05 using Benjamini–Hochberg (BH) correction; SAM: delta = 0.05 with number of permutations = 100; and FCROS: *F*‐value for TopN = 10,000 (cutoff for number of DEGs to be extracted using fvalTopN function). The parameter *N* for FCROS was experimented upon for different values and a suitable value of 10,000 was selected. The results of experimentation are provided in Supplementary Table 1. The pipeline was executed for all three methods to find common DEGs in three cancers at three cutoff levels, log‐FC (LFC)>1 (2‐FC), LFC>1.5 (3‐FC) and LFC>2 (4‐FC). LIMMA produced FC values in a log scale whereas SAM and FCROS produced non‐log FC values. These FC values of LIMMA were hence transformed to non‐log scale so as to be comparable across three methods. Thus, an intersection over the obtained DEG lists could be performed having FC values on the same scale. With three executions of the pipeline for three methods, gene lists using LIMMA (*M*_L1_, *M*_L1.5_ and *M*_L2_), SAM (*M*_S1_, *M*_S1.5_, *M*_S2_) and FCROS (*M*_F1_, *M*_F1.5_, *M*_F2_) were obtained at LFC>1, LFC>1.5 and LFC>2.

#### 2.2.2 RNA‐seq data

**Fig. 2:**
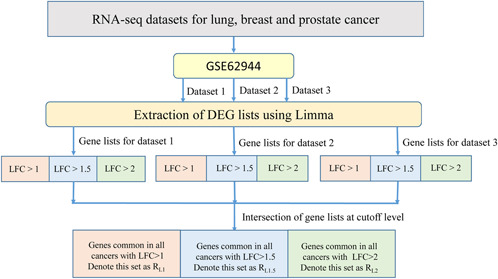
Pipeline for identification of DEGs in three cancer types from RNA‐seq data using LIMMA

LIMMA with *voom* function was chosen to formally identify DEGs from RNA‐seq data of three cancers (*P*‐value<0.05 with BH correction). Normalisation function *voom* is introduced in LIMMA R package specifically for RNA‐seq data. It performs a locally weighted scatterplot smoothing (LOWESS) regression to translate the mean‐variance trend into precision weights using same linear modelling as for microarrays. Benefits of using LIMMA are best explained in [30]. It was shown that LIMMA had comparable, and by some measures improved performance to the other models, which were adapted for RNA‐seq analysis. The advantages include low number of false positives, high correlation between signal‐to‐noise ratio versus *P*‐value for genes detected in one condition, support for multi‐factored experiments and low runtime. It was shown that LIMMA demonstrates close to ideal modelling and is well suited for detecting DE genes. Moreover, it has a capability to analyse both RNA‐seq and microarray data with very similar pipelines [29]. Fig. 2 shows complete pipeline for DEG extraction at three FC cutoff levels. Thus, three gene lists (*R*_1_, *R*_1.5_ and *R*_2_) comprising common DEGs in three cancers were obtained at LFC>1, LFC>1.5 and LFC>2.

#### 2.2.3 Collective DEG sets shared by microarray and RNA‐seq

To obtain consistent DEG sets across three cancer conditions, DEG lists obtained from analysis of each cancer at three FC cutoff levels were evaluated. The gene lists evident from microarray analysis using LIMMA, SAM and FCROS were intersected upon with the gene lists obtained from RNA‐seq analysis. Multiple union and intersection operations were performed on the DEG lists, to agree upon the most consistent set of DEGs prevalent in the cancers.

### 2.3 FC based construction of PPI network

To observe the effect of 2‐FC (LFC>1), 3‐FC (LFC>1.5) and 4‐FC (LFC>2) in expression of significant genes over regulation of cancer pathways and cellular functions at the protein level, three different PPI networks were constructed. STRING interactions (confidence score>500) for gene list with LFC>1, LFC>1.5 and LFC>2 were used to construct the networks. NetworkAnalyst (http://www.networkanalyst.ca) was used for this purpose [31]. The topological analysis was conducted further to identify hub genes based on degree and betweenness centrality (BC) of the nodes. Lists of significant hub genes from all the networks were combined to construct a common cancer network involving prime interactions. This network was further analysed topologically to distinguish hub genes. Functional enrichment analysis was conducted to verify their association with three cancers. Additionally, communities with different clique sizes were plotted and studied.

## 3 Results and discussion

### 3.1 Overlap of gene signature across microarray and RNA‐seq results

**Fig. 3:**
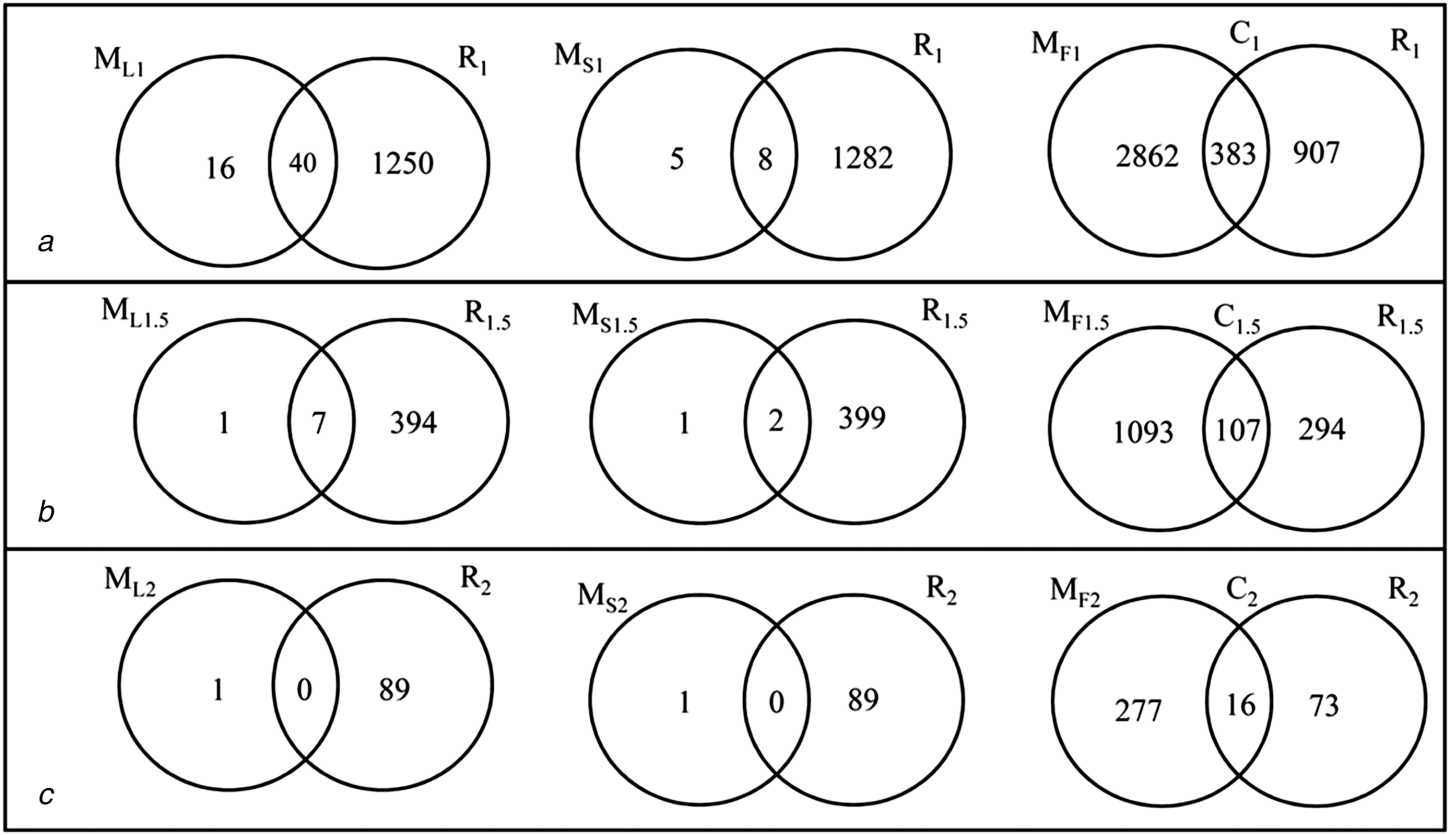
Overlap of DEGs between microarray and RNA‐seq results ***(a)*** Overlap of gene lists at LFC>1, ***(b)*** Overlap of gene lists at LFC>1.5, ***(c)*** Overlap of gene lists at LFC>2

From microarray data analysis nine lists of DEGs (three from each method), and from RNA‐seq data analysis three lists of DEGs were obtained. The number of DEGs in all individual lists is given in Supplementary Table 2. In order to extract gene list evident from both data platforms and to compare the results with respect to number of DEGs obtained using different analysis methods, we performed an intersection of the obtained gene list. The results are illustrated in Fig. 3. Strong concordance was observed between microarray FCROS and RNA‐seq results. It was also important to observe from Fig. 3*a* that eight DEGs from $M_{S1}\cap R_{1}$ were a subset of 40 DEGs from $M_{L1}\cap R_{1}$. This was also true for the results of Fig. 3*b*. This proved that DEG lists using LIMMA were a superset of DEGs from SAM. At the same time, to compare the lists from LIMMA and FCROS, 40 genes from $M_{L1}\cap R_{1}$ were a subset of list of 383 genes from list $C_{1}$ of Fig. 3*a*. Similarly, seven genes from $M_{L1.5}\cap R_{1.5}$ were a subset of 107 genes from list $C_{1.5}$ of Fig. 3*b*. These subsets were obtained as a result of intersection operation performed over the sets and the results are shown in Supplementary Fig. 1. This proves that FCROS is able to capture a superset of DEGs from microarray platform among the three cancer types. Also, no significant DEGs were observed from microarray data at LFC>2 using LIMMA and SAM, whereas FCROS could capture 16 genes which were common to RNA‐seq platform. This list is denoted as $C_{2}$ shown in Fig. 3*c*.

### 3.2 Construction of common cancer network and identification of NMs with FC implications

**Fig. 4:**
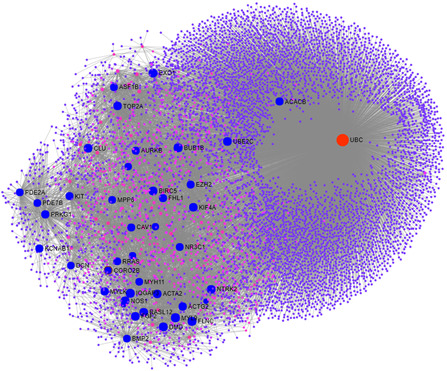
$Network_{FC1-1.5-2}$ *obtained from combining hub genes of* $Network_{FC1}$, $Network_{FC1.5}$ *and* $Network_{FC2}$

Effect of level of FC of the identified common DEGs was studied at protein level by constructing PPI network. Three gene sets $C_{1}$, $C_{1.5}$ and $C_{2}$ for LFC>1, LFC>1.5 and LFC>2 were chosen to construct three PPI networks using NetworkAnalyst tool. Since $C_{1}⊃C_{1.5}⊃C_{2}$, interactions of 276 genes of $C_{1}$, 91 genes of $C_{1.5}$ and 16 genes of $C_{2}$ were extracted from the STRING database [32]. For 276 genes, a PPI network with 3162 nodes‐5371 edges (denoted as $\text{Networ}k_{\text{FC}1}$), for 91 genes, network with 1801 nodes‐2468 edges (denoted as $\text{Networ}k_{\text{FC}1.5}$) and for 16 genes, network with 82 nodes and 83 edges (denoted as $\text{Networ}k_{\text{FC}2}$) were obtained. The corresponding PPI networks are illustrated in Supplementary Figs. 2–4, respectively. Degree and BC of each of the nodes was measured to perform topological analysis of the three networks. BC was considered to be important as an indication of its occurrence in large number of shortest paths between the nodes, which in turn points towards its involvement in many biological processes and functions. With large number of nodes in $\text{Networ}k_{FC1}$, nodes with high degree (≥40) were selected. These nodes were also observed to show high BC values. Similarly, hubs with degree ≥10 were selected from $\text{Networ}k_{\text{FC}1.5}$ and hubs with degree ≥5 were selected from $\text{Networ}k_{\text{FC}2}$, and their BC values were recorded. The list of these genes along with their degree and BC values is provided in Supplementary Table 3. Combined list of hubs from both networks highlighted 81 genes (after removing duplicates) which were used to construct a cancer network of hub genes with FC implications. This was denoted as $\text{Networ}k_{\text{FC}1-1.5-2}$ with 7199 nodes‐11,787 edges. The network is shown in Fig. 4.

Topological analysis of this network was conducted to identify hubs with degree ≥10 which resulted in a list of 108 significant markers. It was important to note that *UBC* was the node with highest degree (6020) and highest BC (25064309) values. Also, it was the only common interacting hub between the nodes of $\text{Networ}k_{\text{FC}1}$, $\text{Networ}k_{\text{FC}1.5}$ and $\text{Networ}k_{\text{FC}2}$. Due to this interconnection between the nodes of the three FC networks through *UBC*, the BC values of the hub genes of all three networks was seen to increase in the combined network. Fig. 4 highlights *UBC* in red and all other hub nodes in blue. The small pink nodes are those nodes whose degree is comparatively small as compared to the hub nodes in the network. Also, the small purple nodes are those nodes whose degree is one, i.e. these are kind of leaf nodes of the graph. The impact of level of FC of DEGs is postulated at the protein level in this network by the degree of interactions shared by the hub nodes. As this network points towards the significant interacting partners of the FC‐based DEGs, it discovers a set of NMs sharing a previously unknown relation at the protein level. This interconnection between the NMs at varied FC levels may be responsible for decision making, triggering of biological processes, regulation of cancer pathways or execution of phenotype.

### 3.3 Significance evaluation of $\text{Networ}k_{FC1-1.5-2}$ in cancer

The cancer network obtained was evaluated with respect to known cancer genes, functional enrichment analysis and complete subgraph enrichment within communities. The findings adhere to the predictions of this network showing high relevance to cancer. The observations are as follows.

#### 3.3.1 Overlap with known cancer genes

**Fig. 5:**
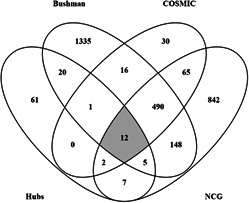
Overlap of NMs with known cancer gene lists where 12 NMs (KIT, EZH2, MYH11, BUB1B, HSP90AA1, AKT1, EGFR, RHOA, HRAS, HSP90AB1, MAPK1, H3F3A) are known cancer genes

Three known cancer genes gene lists were downloaded from Bushman Lab [33], COSMIC [34], NCG [35], and an overlap of our 108 NMs was performed. The results are shown in Fig. 5. It is observed that all three lists comprised of different oncogene sets agreeing upon 12 of our NMs. Similarly, 35 NMs were already known, while 61 are reported to be novel. The list of genes for all the overlaps is provided in Supplementary Table 4.

#### 3.3.2 Complete subgraphs using CFinder

Cliques or complete subgraphs in PPI networks help in predicting protein complexes and functional modules that are involved in important biological processes. Hence, our network was also evaluated for finding cliques involved in different communities detected by CFinder [36]. The interaction of $\text{Networ}k_{\text{FC}1-1.5-2}$ was given as input to determine all significant communities for various clique sizes. The results are illustrated in Table 1. A different number of cliques were obtained in communities for different clique size (*k*). We find the occurrence of gene *UBC* in all the cliques within each community, so as to ascertain its importance in all complete subgraphs within the network. It was found in 3143 cliques out of the total 3936 cliques, which emphasises its functional utility in the interaction between nodes at different FC levels.

**Table 1:** Results of number of communities based on clique size by CFinder

Clique size (*k*)	Number of communities	Community number (number of nodes)	Number of cliques	Number of cliques where UBC appears
6	1	0 (20)	15	15
5	3	0 (71)	84	59
		1 (15)	11	11
		2 (5)	1	1
4	6	0 (227)	285	214
		1 (7)	4	4
		2 (128)	240	239
		3 (33)	29	29
		4 (14)	13	3
		5 (9)	18	0
3	4	0 (1871)	3200	2568
		1 (36)	34	0
		2 (3)	1	0
		3 (3)	1	0
total			3936	3143

#### 3.3.3 Gene Ontology (GO) functional enrichment

Functional enrichment analysis of all the nodes of $\text{Networ}k_{\text{FC}1-1.5-2}$ was performed using GO. Significant biological processes (BP), cellular components (CC) and molecular functions (MF) with large number of hits and *P*‐value<0.05 were selected by the NetworkAnalyst tool. Enriched pathways were also mapped from the KEGG database using KEGGMapper tool (http://www.genome.jp/kegg/mapper.html). Details of all BP, CC, MF and KEGG are given in Supplementary Table 5. Some important pathways indicating relevance of the genes to cancer are listed in Table 2. The shortlisted genes were found to be evident in thyroid cancer, bladder cancer, pancreatic cancer and endometrial cancer other than the three cancers of study which greatly indicate the potential association of NMs with multiple cancer types. Also, the most hit pathways were *metabolic pathway* and *pathways in cancer*. Dysregulation of other significant pathways like MAPK signalling pathway, cell cycle, ErbB signalling pathway, apoptosis and p53 signalling pathway are well defined in cancer [37–41].

**Table 2:** Significant enriched KEGG pathways by Network_FC1‐ 1.5‐ 2_

Pathway	Hits
metabolic pathways	262
pathways in cancer	248
MAPK signalling pathway	144
PI3K‐Akt signalling pathway	140
proteoglycans in cancer	112
microRNAs in cancer	97
breast cancer	69
apoptosis	64
gastric cancer	64
colorectal cancer	51
prostate cancer	50
ErbB signalling pathway	49
small cell lung cancer	48
pancreatic cancer	45
p53 signalling pathway	39
endometrial cancer	32
bladder cancer	26
thyroid cancer	23

## 4 Conclusion

High‐throughput sequencing technologies like RNA‐seq are rapidly replacing microarray technology. However, analysis of both data platforms resulted in detection of crucial and valid gene sets, independent of any technological bias. With the use of multiple DEG extraction methods, the result inconsistency issue has been reduced to minimal. Power of empirical Bayes using LIMMA remains to be authentic as it produced concordant results for both data platforms. However, FCROS produced DEGs based on ranks of FC and showed good agreement to results of LIMMA from microarray and RNA‐seq analysis. Thus, a consistent result set in terms of DEGs from two data platforms was identified. PPI network constructed from FC‐based analysis determined NMs which show high relevance to cancer. The results are distinguished as they reveal significant relation in terms of interconnection between hubs at different FC levels. The presence of *UBC* showing the connection between the hubs of two FC networks points towards the underlying genetic alterations and molecular dynamics common in multiple cancer types. Our approach of using FC‐DEG lists at protein level for evaluation of NMs points towards the need to study relations among the genes. These relations may have a significant biological perspective in the pathogenesis of the disease. It may also have a direct association to the protein complexes and modules formed for the synthesis of important molecular functions. Hence, our approach for FC‐based identification of significant genes in terms of DEGs at genetic level and NMs at protein level provides deep insight to understand complexities common to three cancer types. In the recent decade, various experimental and computational models have been designed to identify novel lncRNA‐disease associations and miRNA‐disease associations [42–44]. It is also shown that miRNAs can function as oncogenes or tumour suppressors in various types of cancers. Similarly, mutations and dysregulations of lncRNAs are associated with the development and progression of different cancer types, including the three cancers of study [45, 46]. With this background, our approach can be extended further for computational modelling to predict novel cancer markers and to establish FC‐based expression and regulation relationships between genes, miRNAs and lncRNAs with the known disease‐gene associations obtained from our results.
